# Evaluation of Chromosomal Structural Anomalies in Fertility Disorders

**DOI:** 10.3390/medicina57010037

**Published:** 2021-01-04

**Authors:** Danielius Serapinas, Emilija Valantinavičienė, Eglė Machtejevienė, Agnė Bartkevičiūtė, Daiva Bartkevičienė

**Affiliations:** 1 Department of Family Medicine, Lithuanian University of Health Sciences, LT-50161 Kaunas, Lithuania; dserapinas@gmail.com (D.S.); e.puisyte@gmail.com (E.V.); 2 Institute of Psychology, Faculty of Human and Social Studies, Mykolas Romeris University, LT -08303 Vilnius, Lithuania; 3 Department of Obstetrics and Gynaecology, Lithuanian University of Health Sciences, LT- 44307 Kaunas, Lithuania; egle.machtejeviene@lsmuni.lt; 4 Clinic of Infectious Diseases and Dermatovenerology, Institute of Clinical Medicine, Faculty of Medicine, Vilnius University, LT-03101 Vilnius, Lithuania; agnebartkeviciute@gmail.com; 5 Clinic of Obstetrics and Gynecology, Institute of Clinical Medicine, Faculty of Medicine, Vilnius University, LT-03101 Vilnius, Lithuania

**Keywords:** chromosomal mutations, infertility, genetic counselling

## Abstract

*Background and objectives:* Reproductive disorders may occur not only due to environmental factors (air pollution, stressful lifestyle, previous abortions or the use of contraceptives) but also due to genetic factors. *Materials and Methods:* The aim of the study was to identify the range and frequency of chromosomal aberrations in couples (*n* = 99) with infertility or recurrent miscarriages in Lithuania. The data were collected from the out-patient medical histories. The couples were divided into three groups based on pregnancy, childbirth and the number of miscarriages. The Chi-square test was used to carry out the statistical analysis, and the statistical significance was (*p* < 0.05). *Results:* There were 6.6% (*n* = 13) structural changes observed in the karyotype tests. Chromosomal aberrations were found in 3% (*n* = 6) of the subjects, while 3.6% (*n* = 7) of them had chromosomal length polymorphisms. No difference was found between the aberration frequency in the karyotypes of men and women (*p* > 0.05). The most common aberrations were balanced translocations (23.1%, *n* = 3) which accounted for 15.4% of the reciprocal (*n* = 2) and 7.7% of the Robertsonian type (*n* = 1) of translocations. The most frequent aberrations were found in couples with the inability to conceive (42.9% (*n* = 3), *p =* 0.031). The childless couples and those with recurrent miscarriages showed an aberration rate of 8.2% (*n* = 5), while in the couples with at least one child it was 16.1% (*n* = 5). The group of couples unable to conceive had a significantly higher aberration rate of 28.6% (*n* = 2), *p =* 0.029. Miscarriages in partners’ families accounted for 8.1%. Miscarriages on the female side of the family accounted for 4.5% (*n* = 9), on the male side it accounted for 2.5% (*n* = 5) and on both sides it accounted for 1.1% (*n* = 2). There were no statistically significant differences observed between the female and male sides (*p* > 0.05). The miscarriages observed in the second group of couples (childless with ≥2 miscarriages) were more frequent at 18.1% (*n* = 11), in the third group (having children ≥2 miscarriages) they were less frequent at 12.9% (*n* = 4), while no miscarriages were recorded in the first group of infertile couples. In total, 3% of the identified significant chromosomal aberrations were likely to trigger miscarriages or the inability to conceive. *Conclusions:* In couples with reproductive disorders, chromosomal mutations and chromosomal length polymorphisms were found at similar rates: 3% vs. 3.6%. The highest aberration rate was found in couples that were unable to conceive, a lower one was found in a group with children and ≥2 miscarriages, and the lowest one was found in a childless group of subjects with ≥2 miscarriages. The miscarriage rate in partner families was 8.1%; however, no difference was found between the male and female sides.

## 1. Introduction

Infertility is defined clinically as the biological state in couples who do not achieve pregnancy after 12 months of regular unprotected sexual life [1,2].

Many causes of infertility are well known and widely described. Fertility disorders are a result of various ailments of the reproductive system or infectious diseases. They range from external environmental factors to genetic determinants and are found in both men and women. However, part of the causes most frequently remain unexplained, for example, the recurrent miscarriages that will receive the primary focus in our study.

Miscarriages most commonly occur during the first 12 weeks of gestation, and the leading cause for miscarriages is genetic defects of the embryo [3,4]. Thus, the most common practice is genetic consultation and investigation of parents, i.e., the couples who have experienced miscarriages. One of the key genetic studies is the determination of a karyotype. Recurrent miscarriages (RM) are one of the causes of the couple’s fertility disorders. Not only do they stimulate extensive discussions about the determination of the cause or the options for treatment, but they also take into account the issues of repeated pregnancy outcomes. According to the agreement of the American Society for the Development of Reproductive Medicine [3], two or more aborted pregnancies confirmed by ultrasound or by a histological examination of the terminated pregnancy’s tissue are considered to be a recurrent miscarriage. One miscarriage occurs in about 15–20% of all couples, and about 1–5% of women suffer from recurrent miscarriages in the United States [4,5]. In Europe, if only clinical miscarriages are included, the prevalence of RM is 0.8% to 1.4%; if, however, biochemical losses are included, the prevalence is estimated to be as high as 2% to 3% [6,7]. Unfortunately, there are no clear statistics on the number of miscarriages in Lithuania. According to the statistics of the Hygiene Institute [8], over 25,000 children are annually born in Lithuania; therefore, the approximate number of females with RM should be between 200 and 1250.

According to foreign scholarly literature, the causes of RM are diverse; therefore, they are most commonly categorized into large groups (Table 1) [5,9,10,11]. Our study has mainly focused on the genetic part that comprises only 3% of the entire range of RM causes [12,13]. However, taking into account the improvement and growth of the genetic research potential, this figure can grow. Therefore, it is important to accumulate the experience of how the country tackles RM problems in a timely manner, which is one of the long-term aims of our study.

The data show that even chromosome structural polymorphisms can be related to fertility disorders. Polymorphisms do not impact the phenotype; however, they have been associated with poor spermatogenesis and male infertility [9]. The main type of research applied in providing genetic consultations for couples with fertility disorders is karyotyping. The most commonly examined tissues fall into two categories: (1) parental karyotype and (2) pregnancy tissues, i.e., the karyotype of the lost fetus.

Chromosomal aberrations found in the karyotypes are categorized into two major groups: the first group involves structural changes in chromosomes, while the second one comprises chromosomal numeral abnormalities. The most commonly occurring structural changes are deletions, duplications, inversions and translocations. Mutations in the number of chromosomes can still be subdivided into the changes in the number of sex/autosomal chromosomes and can cause certain genetic syndromes. However, these changes are not included among the subjects in this study [11].

Our study shows that parental chromosomes are most likely to indicate such changes as a balanced translocation, inversion or duplication in chromosome parts, which are most commonly phenotypically not present due to a lack of genetic material. However, chromosomal aberrations with translocations provide most of the data and are associated with miscarriages the most [12,13]. In addition, partners with chromosomal aberrations in the karyotype have a higher risk of recurrent miscarriages than those with the normal karyotype [14].

A review of the most common parental chromosomal anomalies in the scholarly literature shows that the dominant change is the balanced chromosomal translocation. In 3–5% of the couples who suffered from repeated miscarriages, one of the partners had a balanced translocation [15,16]. The frequency of such people in the population is one in 500, or about 0.2% [16,17]. Based on these data, and compared to other countries, our study is likely to find a similar percentage of chromosomal aberrations among couples with fertility disorders.

According to the data obtained by researchers, about half of the early miscarriages (i.e., up to 12 weeks of gestation) and about one third of the second trimester miscarriages occur due to embryo chromosomal abnormalities. Of these, the changes in the chromosomal number comprise up to 86%, the changes in the chromosomal structure comprise up to 6% and mosaicism comprises up to 8% [18,19]. Embryo chromosomal abnormalities resulting in miscarriage may arise due to the nondisjunction of chromosomes or the appearance of supernumerary marker chromosomes (sSMCs) during meiosis. Numeral chromosomal aberrations (most frequently X chromosome monosomy) usually occur due to female meiosis errors [20]. sSMCs are small, structurally abnormal parts of chromosomes (usually originating from chromosome 15 acrocentric parts) that are extra to a normal set of 46 chromosomes. The overall sSMC frequency is 7.5-fold higher in males than in females. During spermatogenesis, such disturbances may not only lead to a decrease in the number of gametes but also to the incidence of chromosomal de novo aberrations that may increase the risk of repeated miscarriages [21].

Previous spontaneous past abortions in the family are most commonly observed in the relatives of the first and second generation and are slightly more frequent on the female side [22], and, according to some authors, the overall ratio of chromosomal anomalies causing miscarriages in females and males is 2:1 [23].

## 2. Materials and Methods

The outpatient case histories of 99 couples were selected and included in the karyotype result analysis after counselling couples with reproductive disorders. The analysis of the data was performed by calculating the subjects as couples (a total of 99 medical records) and individually (a total of 198 persons). The study was approved by the Bioethics Center of the Lithuanian University of Health Sciences on 3 November 2014, Order No. BEC-MF-104.

The data on the number of pregnancies, childbirths and miscarriages were selected from outpatient case histories, the patients’ genealogical trees were reviewed, and the number of miscarriages in the family (the presence of miscarriages in brothers, sisters and parents was estimated) was taken into account.

The category of patients’ grouping was chosen to distinguish the cases and to reveal whether the determination of the level of karyotype was more relevant to one of the groups compared to others. There were three groups: the couples who failed to conceive (infertile couples), the couples who could conceive but miscarried, and those with at least one child who registered for a genetic consultation following recommendations after the past two or more miscarriages. After the selection of subjects, the karyotype research findings obtained from the couples were included for an analysis of all the changes of the chromosomal structure such as chromosomal length polymorphisms and chromosomal aberrations. The detected chromosomal length polymorphisms involved chromosomal changes such as an increase (e.g., 46, XY, 9qh+) or decrease (e.g., 46, XY, 9qh−) in heterochromatin levels and an enlargement of the satellite region (e.g., 46, XY, 22ps+). The mutations comprised changes in the mosaic, translocation and inversion types of chromosomes in the obtained karyotypes. However specific inversion of the 9th chromosome-46, XY,inv(9)(p11;q12) sometimes is assigned to polymorphisms.

The peripheral blood lymphocyte karyotype was investigated in all the couples. In total, 3 mL of peripheral venous blood was taken from each patient. A cytogenetic analysis was carried out based on the phytohaemaglutinin-stimulated peripheral blood lymphocyte cultures. Lymphocyte culturing and GTG-banding were performed following standard protocols as described by the Cytogenetics Laboratory Manual [24,25]. The karyotypes were described according to the International System for Cytogenetic Nomenclature (ISCN 2013). In each patient, twenty cells in the mitosis metaphase were selected for a G-banding karyotype analysis. If there was a small proportion of the chimeric karyotype, 50–100 cells in the mitosis metaphase were selected.

A statistical data analysis was performed using Microsoft Excel and SPSS 22.0 software (IBM Corp., Armonk, NY, USA). The general descriptive statistics methods were used, and the qualitative variables were compared by a Chi-square test. The selected statistical significance level was (*p* < 0.05).

## 3. Results

In all, 99 outpatient case histories of couples were analyzed. The average age of women was 31.10 ± 5.38 years, and that of males was 33.17 ± 6.06 years. Chromosomal structural changes were found in the karyotypes of 6.6% (*n* = 13) of all the subjects under analysis. Only 3% (*n* = 6) had aberrations that could affect RM or the ability to conceive. Of all, 3.6% (*n* = 7) of individuals had chromosomal length polymorphisms that were a normative variant. The overall rate of chromosomal changes in female karyotypes was 4% (*n* = 4), and in men it was 9.1% (*n* = 9). There was no statistically significant difference between the male and female chromosomal aberration rate in the karyotype studies (*p* > 0.05): there were 3% in both sexes (*n* = 3). Chromosomal length polymorphisms were more frequently observed in males (*n* = 6) than in females (*n* = 1).

**Figure 1:**
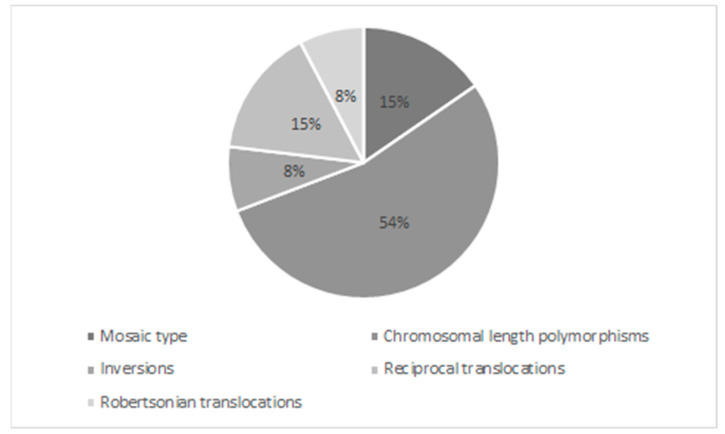
Rates of the distribution of chromosomal rearrangements.

Among all the chromosomal aberrations, balanced reciprocal translocations and mosaic-type aberrations were more frequently observed, at 15.4% (*n* = 2). The Robertsonian type translocation and inversions were the only cases, at 7.7% (*n* = 1) (Figure 1).

The frequency of chromosomal changes between the three groups (Table 2) was as follows: 7.1% (*n* = 7) who were unable to conceive, 61.6% (*n* = 61) who were childless with ≥2 miscarriages, 31.3% (*n* = 31) who were couples having children and had ≥2 miscarriages. The comparison of the chromosomal structure found in the karyotypes between these groups showed that aberrations were more frequent in couples unable to conceive (42.9% (*n* = 3), *p =* 0.031). The aberration rate in the group of couples who were childless with miscarriages was only 8.2% (*n* = 5), and in the group with children it was 16.1% (*n* = 5). The rate of aberrations was also more frequently found in the group of couples unable to conceive, at 28.6% (*n* = 2), *p =* 0.029, when compared to the other two groups (Table 2 and Table 3).

In total, 16 patients (8.1% of all the subjects) were documented with a family history of miscarriages. In all, 4.5% (*n* = 9) of cases with a history of miscarriage were found on the female side only, 2.5% (*n* = 5) were found on the male side, and 1.1% (*n* = 2) of case histories showed that miscarriages occurred in both males and females. There was no statistically significant difference between women and men (*p* > 0.05).

The comparison of the frequency of miscarriages among the three groups of couples revealed that the most frequent miscarriages occurred in the families of the second group (childless with ≥2 miscarriages), at 18.1% (*n* = 11), they were less frequent in the third group (with children ≥2 miscarriages), at 12.9% (*n* = 4), and no miscarriages were found in the first group of couples that were unable to conceive. However, there was no statistically significant difference between the groups.

## 4. Discussion

Our study showed that the chromosomal change rate (including chromosomal length polymorphisms) in the karyotypes of couples was 6.6% and that 3% of the changes were chromosomal aberrations that were likely to affect RM or the inability to conceive. The limitation of our study was that the chromosomal rearrangements were not confirmed by FISH.

These figures are slightly lower than those presented by other countries [26,27]. In terms of the type of chromosomal changes, translocation-type mutations were more frequently found, which supports the data presented by most of the Lithuanian and foreign scholars. Interestingly, a significant proportion (15.4%) of all significant aberrations was found in a mosaic type, which is less frequent in chromosome alterations. With reference to the literature, a mosaic karyotype is a rare change in subjects with a past history of RM [28], but it is quite commonly found in the karyotyping of miscarriage tissues. For example, according to Vorsanova et al., mosaic types were even observed in 48.3% of cases of karyotyping of miscarriage tissues [29]. Chromosomal aberrations were more frequently seen in couples who consulted for the inability to conceive rather than in other groups of couples. Taking into account that this group was the smallest, the statistical data may deviate from the actual situation in the general population. In terms of consulting couples concerned with infertility, genetic consultations and the determination of the karyotype should be one of the stages of the investigation, and the data we have received confirm the need for genetic testing. Furthermore, the determination of the parental karyotype for infertile couples is also recommended in cases where assisted fertilization techniques such as in vitro fertilization are opted for [30]. Even though the determination of a karyotype may not seem desirable in couples with children since the presence of children already shows the ability of a couple to have offspring, our study found that even in the group of couples with children and with two or more miscarriages, the chromosomal aberration rate was 16.1%, which was twice as frequent as for the group of childless couples with two or more miscarriages (8.2%). These findings indicate that the number of miscarriages is a more important indicator than the number of children that are born. Therefore, it is currently recommended that one perform karyotyping for all couples with a history of two or more miscarriages.

Having assessed the documented miscarriages in the family, we found that they were more frequent on female (4.5%) than on the male (2.5%) side and that 1.1% of them were observed on both partners’ sides. Summing up the results, we cannot argue that a couple has a higher risk of having an RM with a female family with miscarriages, as no statistically significant difference between the sexes has been obtained. In addition, part of the past miscarriages in the family may not have been documented due to the patients’ lack of knowledge about the health of their parents or their brothers and sisters. Thus, the received data may not reflect the actual situation. To achieve this, the scope of the study should be expanded, and a careful assessment of the family history should be made. The scholarly data on the frequency of pregnancy interruptions in the family for couples with RM are insufficient in both the Lithuanian and foreign literature. One of the largest studies was carried out in the Netherlands [31], where a miscarriage in the family (if brothers, sisters or parents had suffered from at least two or more miscarriages) was seen as one of the risk factors in partners finding chromosomal changes in the karyotype. Therefore, based on our findings and the literature review, only one miscarriage among the partner families was unlikely to be a cause for concern.

In genetic counselling for fertility problems before administering cytogenetic or other necessary molecular research, the past history is a major issue not only in assessing the number of past miscarriages, birth defects or inherited illnesses, but also in evaluating other potential risk factors. For example, older age, overweight and harmful habits (mostly smoking and alcohol consumption) are considered to be risk factors for a miscarriage [32,33]. If possible, the risk factors should be corrected in the first place, and only then can expensive cytogenetic studies be considered.

In counselling couples with fertility problems, karyotype testing is not absolutely necessary. Having assessed each case on an individual basis, further molecular genetic studies are possible. Some of the most common causes accounting for miscarriages, as observed in the clinical practice of a geneticist, are the Leiden V factor, prothrombin, MTHFR gene mutation, antiphospholipid syndrome, and various autoimmune diseases associated largely with HLA—human leukocyte antigens. Taking into account that the disclosure of the frequency of various gene mutations for couples with fertility disorders was not the purpose of our study, we refrain from further development of these issues and leave them as the subject for further studies.

In summary, the karyotyping of couples with fertility disorders is an important part of a genetic consultation because, depending on the outcome of the response, it is possible to predict the progress of further pregnancies and consider family support. For example, according to the literature data, if one of the partners is a carrier of a balanced translocation, approximately two-thirds of couples succeed in having a child [34]. Depending on which chromosome is included in aberrations, the probability of a child with a genetic syndrome can be estimated. The most commonly mentioned example is where a carrier of a 21-chromosome balanced translocation transmits a change in the child, causing Down syndrome (21 chromosome trisomy). If the carrier is a woman, according to the literature, the probability of having a child with Down syndrome is about 10%, while in the case of a man-carrier it is 0.5% [35]. On the other hand, in such cases the patients should be advised to undergo a prenatal diagnosis for other pregnancies.

## 5. Conclusions

The chromosomal aberrations rate that may affect RM or the inability to conceive is 3%. The balanced translocations and mosaic aberrations were most frequent. Chromosomal aberrations were more common among couples unable to conceive (*p* < 0.05) when compared to other groups of couples (with miscarriages). In total, 8.1% of the cases suffered from miscarriages in the family. There was no significant difference regarding whether more frequent miscarriages (*p* > 0.05) occurred on the male or female side.

## Figures and Tables

**Table 1:** Overview of miscarriage-associated factors and their possible causal role in recurrent miscarriages.

Etiology	Percentage (%) [Ref.]
Immune	25 [5]
Anatomical	22 [2,6]
Endocrinological	20 [13]
Microbiological	6 [7]
Genetic	3 [9,11,12]
Environmental factors	40 [7,10]

**Table 2:** Rearrangement of the chromosomal structure in different groups of couples.

Groups of Couples	Unable to Conceive	Childless with ≥2 Miscarriages	Having Children with ≥2 Miscarriages	In Total: % (*n*)
Individuals	(*n* = 14)	(*n* = 122)	(*n* = 62)	(*n* = 198)
Normal karyotype	57.7%	91.8%	83.9%	93.4%
	(*n* = 11)	(*n* = 117)	(*n* = 57)	(*n* = 185)
Rearrangements, all together	42.9% *	8.2%	16.1%	6.6%
	(*n* = 3)	(*n* = 5)	(*n* = 5)	(*n* = 13)
Chromosomal aberrations	28.6%	3.3%	6.5%	3%
	(*n* = 2)	(*n* = 2)	(*n* = 2)	(*n* = 6)
Chromosomal length polymorphisms	14.3%	4.9%	9.6%	3.6%
	(*n* = 1)	(*n* = 3)	(*n* = 3)	(*n* = 7)

Chromosomal aberrations included: reciprocal and Robertsonian translocations, mosaicism, inversion. * *p* < 0.05 between three groups of couples.

**Table 3:** Description of the structural chromosomal changes found in the karyotypes of the subjects.

Chromosomal Aberrations	
Inversion	46,XY,inv(9)(p11;q12)
Reciprocal translocations	46,XY,t(1;8)(p31;p22)46,XX,t(10;11)(p12;q22)
Robertsonian translocation	45,XX,der(13;14)(q10;q10)
Mosaic karyotype	mos 47,XX,+mar (5)/46,XX (65) mos 47,XY,16qh+,+mar (5)/46,XY,16qh+ (45)
**Chromosomal length polymorphisms**	
Heteromorphisms	46,XY,9qh+ (3 cases)
	46,XY,16qh−
	46,XY,9qh−
Enlargement of the satellite region	46,XX,13ps+ 46,XY,22ps+

Inv—inversion, t—translocation, der—derivate, mos—mosaicism, h—heterochromatin, ps—satellites.

## Data Availability

The data are not publicly available due to genetic information privacy protection.
